# Modeling and Prediction of the Covid-19 Cases With Deep Assessment Methodology and Fractional Calculus

**DOI:** 10.1109/ACCESS.2020.3021952

**Published:** 2020-09-04

**Authors:** Ertuğrul Karaçuha, Nisa Özge Önal, Esra Ergün, Vasil Tabatadze, Hasan Alkaş, Kamil Karaçuha, Haci Ömer Tontuş, Nguyen Vinh Ngoc Nu

**Affiliations:** 1 Informatics Institute, Istanbul Technical University 52971 34467 Istanbul Turkey; 2 Faculty of Society and EconomicsRhine-Waal University of Applied Science 47533 Kleve Germany; 3 Faculty of Science and LettersIstanbul Technical University 52971 34000 Istanbul Turkey

**Keywords:** COVID-19, deep assessment methodology (DAM), fractional calculus, least squares, long short-term memory, modeling, prediction of pandemics, SIR model

## Abstract

This study focuses on modeling, prediction, and analysis of confirmed, recovered, and death cases of COVID-19 by using Fractional Calculus in comparison with other models for eight countries including China, France, Italy, Spain, Turkey, the UK, and the US. First, the dataset is modeled using our previously proposed approach Deep Assessment Methodology, next, one step prediction of the future is made using two methods: Deep Assessment Methodology and Long Short-Term Memory. Later, a Gaussian prediction model is proposed to predict the short-term (30 Days) future of the pandemic, and prediction performance is evaluated. The proposed Gaussian model is compared to a time-dependent susceptible-infected-recovered (SIR) model. Lastly, an analysis of understanding the effect of history is made on memory vectors using wavelet-based denoising and correlation coefficients. Results prove that Deep Assessment Methodology successfully models the dataset with 0.6671%, 0.6957%, and 0.5756% average errors for confirmed, recovered, and death cases, respectively. We found that using the proposed Gaussian approach underestimates the trend of the pandemic and the fastest increase is observed in the US while the slowest is observed in China and Spain. Analysis of the past showed that, for all countries except Turkey, the current time instant is mainly dependent on the past two weeks where countries like Germany, Italy, and the UK have a shorter average incubation period when compared to the US and France.

## I. Introduction

Public health programs and capacity planning are essential for tracing and managing health risks. These programs focus on prevention more than diagnosing, detection, and responding to the cases, including infectious disease outbreaks [1]. To plan successful preventive activities, solid estimates are needed about the spread and localization of the disease, and the possible path and case numbers. Therefore, reliable mathematical predictive modeling for any kind of diseases (especially for communicable diseases) is an essential tool for public health policies. European countries were not particularly successful in combating the COVID-19 outbreak due to the insufficiency of such predictions.

The spread of COVID-19 could not be effectively avoided at its origin and has become a pandemic that infects more than 8 million people in 215 countries and territories as of 2020 [2]. It is important to understand the behavior of the pandemic in order to prepare health care workers and other related parties. The modeling and prediction of COVID-19 have crucial importance for planning hospital resources [3]. To combat with further progression of the COVID-19 infection requires predictive modeling. The authorities can take required precautions regarding the staff and/or hospital resources to overcome the epidemic-like difficulties they may encounter in the future by predicting the progression of the infection [3].

Recent developments in digital technology and informatics in parallel with the development of data science lead the companies, institutions, universities and especially, the countries to give priority to evaluating the data and predicting what can be forthcoming [3]. Computational mathematical methods have a significant role in understanding the dynamics of an epidemic and application of such methods on biological systems has been an interest to many researchers throughout decades [4]–[7]. Because of the current outbreak of COVID-19, many mathematical methodologies are being investigated on predicting the trend, estimating the peak, and modeling the course of the pandemic. The models can be used separately or in combination, [8] adopts three types of mathematical models for fitting COVID-19 data: the logistic model, Bertalanffy model, and Gompertz model. The least-square method is used for fitting and the final cumulative number of confirmed cases is predicted. Another study, [9], combines ARIMA and wavelet-based predicting techniques for 5 countries. In [10] a method to predict the spread of COVID-19 based on dictionary learning and online nonnegative matrix factorization is proposed and evaluated for one-step predictions and extrapolated near future. In [11], a stochastic transmission dynamic model is fitted to multiple publicly available datasets by using sequential Monte Carlo simulation and resulting number of cases, transmission rate over time, and basic Reproduction number ($R_{t}$) is inferred. Besides, Schüttler *et al.* [12] proposed a Gauss Model to map time to the Gauss function to model the daily deaths per day and country. In [13], authors show that simple mean-field models can be used to interpret epidemic spreading and to forecast time of the peak of confirmed cases. Another mathematical method based on the data analysis of logistic growth equations describing the process on the macroscopic level is used in an attempt to calculate the expected end of the outbreak in Italy [14]. Also, [15] carries out a time series analysis using a mixed-effect model of lagged, log-linear case counts for each province and predicts a province-level growth rate in China.

Another well-known model that is widely applied by researchers is the susceptible-infected-recovered (SIR) model [16]–[18] which uses mainly three variants of infection: susceptible, infected, and recovered. Nesteruk [19] used the SIR model to predict the epidemic characteristics in mainland China while Batista [20] applied it to estimate the final size of the Covid-19 epidemic. However, in the original SIR model transmission rate $\beta $ and recovering rate $\gamma $ remain as time-invariant variables. As a result, such models are unable to include the impacts of specific state interventions to control and avoid the spreading of the virus in different periods into the SIR-modelling. Therefore, Ping-En Lu used an extended version of SIR called Time-dependent SIR model, in which both transmission rate $\beta $ and recovering rate $\gamma $ are also functions of time so that model could adapt and adequately predict the trend of COVID-19 in China [21]. Before that, the SIR epidemic model had also been used to describe the spread of other diseases such as influenza and measles [22], [23].

One of the mathematical approaches to model a physical phenomenon is setting up a differential equation where the dependent variable satisfies a differential equation with respect to the independent variable. Integer order differential equations cannot model processes with memory and non-locality because of their locality property. On the other hand, Fractional Calculus is a branch of mathematics that focuses on fractional-order differential and integral operators and can be used to address the limitations of integer-order differential models. Fractional operators convert the integer-order differential equations into the non-integer order differential equation and lead to a very essential advantage: the memory property. Fractional order derivatives are the generalization of the integer order counterparts and represent the intermediate states between two known states. For example, zero order-derivative of the function represents the function itself while the first-order derivative represents the first derivative of the function. Between these known states, there are infinitely many intermediate states [24]. Therefore, fractional operators provide more accurate models in many branches of science and engineering including mechanics, biology, biomedical devices, nanotechnology, diffusion, diffraction, and economics [25]–[43].

Memory property of fractional calculus has been employed for modeling and prediction on epidemics such as Measles, Malaria, Dengue, and Ebola [44]–[47]. Recently, [48] illustrated that the memory feature of the fractional derivative explores the hidden dynamics of infection in contrast to an integer type of derivative by analyzing the data of India. Furthermore, in our previous studies, methods based on fractional calculus (FC) that work for modeling and prediction of time series were introduced. In these studies, the children’s physical growth, subscriber numbers of operators, GDP per capita were modeled and compared with other modeling approaches such as Fractional Model-1 and Polynomial Models [3], [7], [49], [50]. According to the results, proposed fractional models had better results compared to the results obtained from Linear and Polynomial Models [3], [7], [49], [50]. As a result, the existing applications of the fractional differential approach on various biological studies and success of our prior work on modeling and prediction of time series using fractional approach are the foundations of our motivation to analyze COVID-19 with fractional calculus.

This study focuses on modeling and predicting the trend of COVID-19 pandemic for eight countries including China, France, Germany, Italy, Spain, Turkey, the UK, and the US. The first main focus of this study is modeling and the one-step prediction of the pandemic. After modeling and having the mathematical expression for the dataset, a short-term future (30-Days) prediction is investigated. In this study, we mainly apply our “Deep Assessment Methodology (DAM)” [3] for predicting the case numbers and death numbers related to the coronavirus pandemic. DAM is a method that is derived from the fractional differential equations for modeling and prediction purposes and uses the properties of fractional calculus. The method utilizes the corresponding Laplace transform properties. Here, the data is modeled with an approach that considers the effect of a finite number of previous values and the derivatives. Further, the prediction is obtained by assuming a value in a specific time can be expressed as the summation of the previous values weighted by unknown coefficients and the function to be modeled is continuous and differentiable. In this way, DAM takes previous values and variation rates between different time samples (derivative) of the dataset into account while modeling the data itself and predicting upcoming values. Combining the previous values with the variations weighted by the unknown coefficients lead to calling this method as “deep assessment”. There are two advantages of the method regarding other approaches mentioned in this section. First, the proposed method uses the generalization of the derivative operator. The generalization of the derivative operator provides flexibility when modeling the structure of the data. In this way, the modeling has one more parameter to control and optimize the error between the proposed modeling and real data. Second, the deep assessment method has the ability of both modeling and prediction. During modeling, the present value is expressed as a summation of the previous values and changes of the function with unknown weights, which is then optimized by the Least Squares Method. Thus, combining the fractional derivative and defining the instant value as the summation of the weighted previous values and changes, the prediction can be obtained since the epidemic can be assumed as a system with memory. In other words, previous values and the increase between the intervals can affect the present and the future. To overcome the limitation of the locality which presents in integer-order derivates, the fractional differential equation is utilized which includes the hereditary and non-locality properties of the non-integer order derivatives.

In a recent study [51], a mathematical model is presented by incorporating the isolation class to model the dynamical behavior of COVID-19. Their model employs a Nonstandard Finite Difference (NSFD) scheme and Runge-Kutta fourth-order method. In [52], outcomes of various models such as SIR, SEIR, and ARIMA are discussed. Further, [53] develops two models to capture the trend of dynamic: A mathematical model that accounts for various parameters related to the spread of the virus and a non-parametric model that uses the Fourier Decomposition Method (FDM).

The number of confirmed cases (prevalence), the intensity of cases in a specific location (incidence), and the number of deaths can be used as a proxy of the effectiveness of a country’s fight against the pandemic. In general, it is a reasonable measurement of a country’s health system strength and public health strategy against COVID-19 [54]. Some researchers used the date of the first recorded case and cumulative case numbers as a criterion for evaluating coronavirus pandemic in certain countries [1], [2]. Therefore, the prediction of case numbers and death numbers is very essential not only for the researchers but also for governments and institutions to take action against an uncontrolled spreading. The reliable number of case predictions defined by modeling based on scientific foundations is vital for the relevant institutions and organizations to determine their road maps.

As shown in our previous study [3], DAM successfully models and predicts time series data. In this study, we predicted the total number of confirmed cases, death, and recovered case numbers for 8 countries including China, France, Germany, Italy, Spain, Turkey, the UK, and the US using our previously proposed work, Deep Assessment Methodology. To assign performance of DAM on the COVID-19 dataset we compared the model with Long Short-Term Memory (LSTM), a special type of artificial neural network used in analyzing time series problems. Later, a Gaussian fitting approach based on DAM is proposed for predicting the short-term future of the pandemic and compared to the Time-dependent SIR model [21]. Lastly, an analysis of model coefficients is made with wavelet denoising and correlation coefficients to understand the effect of the past.

The structure of the study is the following. Section II explains the Formulation of the Modeling Approaches where two methods that utilize fractional calculus are introduced. After that, Section III, namely the Proposed Approaches, are devoted to explaining how to obtain modeling, simulation, testing, and prediction. In this section, Deep Assessment Methodology, the prediction with Deep Assessment, Time-dependent SIR model, Modeling Based on Gaussian Distribution with DAM, and Prediction with LSTM are explained. Then, in Section IV, the results are presented and compared. Section V discusses the limitations of the study. Lastly, Section VI highlights the conclusion of the study.

## II. The Proposed Approaches

### A. Modeling and Prediction with Deep Assessment Methodology

This section is dedicated to our previously proposed modeling and prediction approach Deep Assessment Methodology (DAM) [3]. The two main goals of DAM are to find a function representing the dataset optimally and to predict the unknown upcoming values. To achieve these goals, DAM exploits fractional calculus and Taylor series expansion. It is suggested for the readers to see more detail by referring [3].

To express or infer an event or a phenomenon that depends on its previous values and states, the effects of previous values and change over time on the current time instant should be understood. With this motivation, DAM represents a function $g(x)$ with finite summations of its previous values and derivatives of the previous values weighted with unknown coefficients $\tilde {\alpha }_{k}$ and $\tilde {\beta }_{k}$, as shown in (1).

\begin{equation*} g\left ({x }\right)\cong \sum \limits _{k=1}^{l} \tilde {\alpha }_{k} g\left ({x-k }\right)+\sum \limits _{k=1}^{l} \tilde {\beta }_{k} g^{\prime }\left ({\text {x-k} }\right)\tag{1}\end{equation*}

Here, $g^{\prime }$ stands for the first-order derivative of $g\left ({\text {x-k} }\right)$ with respect to $x$. Approximating the function in such a way leads to achieving the memory property. Later, assuming that $g\left ({x }\right)$ is a differentiable and continuous function, DAM expands $g\left ({x }\right)$ as Taylor Series with unknown constant coefficients $\tilde {a}_{n}$’s as given in (2).

\begin{equation*} g\left ({x }\right)=\sum \limits _{n=0}^\infty \tilde {a}_{n} x^{n}\tag{2}\end{equation*}

Similarly, a previous time instant is expressed as

\begin{equation*} g\left ({\text {x-k} }\right)=\sum \limits _{n=0}^\infty \tilde {a}_{n} {(x-k)}^{n}\tag{3}\end{equation*}

Using (1) and (3), the form of the function $g\left ({x }\right)$ is obtained as follows.

\begin{align*}&\hspace {-.5pc} g\left ({x }\right)\cong \sum \limits _{k=1}^{l} \tilde {\alpha }_{k} \sum \limits _{n=0}^\infty {\tilde {a}_{n}\left ({\text {x-k} }\right)}^{n} \\&\qquad\qquad\qquad\qquad\displaystyle { +\sum \limits _{k=1}^{l} \tilde {\beta }_{k} \sum \limits _{n=0}^\infty {\tilde {a}_{n}n(x-k)}^{n-1} } \tag{4}\end{align*}

Truncating $\infty $ to $M$ for the numerical calculations and expressing unknown coefficients $\tilde {\alpha }_{k}\tilde {a}_{n}$ as $\tilde {a}_{kn}$, $\tilde {\beta }_{k}\tilde {a}_{n}$ as $\tilde {b}_{kn}$ yield the final form of the approximated $g(x)$ function and its first derivative:

\begin{align*} g\left ({x }\right)\cong&\sum \limits _{k=1}^{l} \sum \limits _{n=0}^{M} {\tilde {a}_{kn}\left ({\text {x-k} }\right)}^{n} \\[3pt]&+\sum \limits _{k=1}^{l} \sum \limits _{n=0}^{M} {\tilde {b}_{kn}n(x-k)}^{n-1} \\ \tag{5}\\[3pt] \frac {dg\left ({x }\right)}{dx}\cong&\sum \limits _{k=1}^{l} \sum \limits _{n=1}^{M} {\tilde {a}_{kn}n} \left ({\text {x-k} }\right)^{n-1} \\[3pt]&+\sum \limits _{k=1}^{l} \sum \limits _{n=1}^{M} {\tilde {b}_{kn}n(n-1)} {(x-k)}^{n-2}\tag{6}\end{align*}

After this step, the next step is to include the heritability property [5], [35], [41]. Therefore, first, the fractional derivative of $g(x)$ function needs to be defined. Throughout the study, Caputo’s description of the fractional derivative is employed as in (7) [18].

\begin{align*}&\hspace {-.5pc} \mathfrak {D}_{x}^{\gamma }g\left ({x }\right)=\frac {d^{\gamma }g\left ({x }\right)}{dx^{\gamma }}=\frac {1}{\Gamma \left ({n-\gamma }\right)}\int \limits _{0}^{x} \frac {g^{\left ({j }\right)}\left ({k }\right)dk}{\left ({\text {x-k} }\right)^{\gamma -n+1}}, \\[3pt]&\qquad\qquad\qquad\qquad\displaystyle { \left ({j-1 < \gamma < j }\right) } \tag{7}\end{align*}

In (7), $\Gamma \left ({1-\gamma }\right)$ is the Gamma function. Here, the fractional derivative is taken with respect to $x$ in the order of $\gamma $ and $g^{\mathrm {(j)}}$ corresponds to the $j^{th}$ order derivative with respect to $x$. The derivative is generalized by changing the first-order derivative in (6) to fractional derivative in the order of $\gamma $ as in (7) [3], [7], [49], [50]. In DAM, $j$ is set to 1, and the fractional-order changes between [0, 1]. Using the fractional derivative operator which is inserted into the fractional differential equation contributes to the hereditary property [3], [7], [49], [50]. Throughout the paper it is assumed that function $f(x)$ stands for the total number of COVID-19 confirmed cases, recovery from the infection, cumulative deaths over time with the order of $\gamma $ is equal to (8).

\begin{equation*} f\left ({x }\right)\cong \sum \limits _{k=1}^{l} \sum \limits _{n=0}^{M} {a_{kn}\left ({\text {x-k} }\right)}^{n} +\sum \limits _{k=1}^{l} \sum \limits _{n=0}^{M} {b_{kn}n(x-k)}^{n-1}\tag{8}\end{equation*}

The function $f\left ({x }\right)$ satisfies the fractional differential equation (9) and models the discrete dataset related to COVID-19 as it is given above for $g(x)$ for a general approach.

\begin{align*}&\hspace {-.5pc} \frac {d^{\gamma }f\left ({x }\right)}{dx^{\gamma }}\cong \sum \limits _{k=1}^{l} \sum \limits _{n=1}^\infty {a_{kn}n} \left ({x-k }\right)^{n-1} \\&\qquad\qquad\qquad\qquad\displaystyle { +\sum \limits _{k=1}^{l} \sum \limits _{n=1}^\infty {b_{kn}n(n-1)} {(x-k)}^{n-2} } \tag{9}\end{align*}

Here, $x$ denotes the time. Note that, different from (6), introducing the variable fractional order between 0 and 1 in the derivative operator at the left-hand side of (6) for $f(x)$ gives a more general, flexible model given in (9) [27]. Also, this brings one additional parameter, $\gamma $, the fractional-order to model the dataset optimally. To solve the differential equation, (9) is transformed into the Laplace domain which converts the equation into an algebraic form. After taking the inverse Laplace Transform of the transformed algebraic equation, we get the final form of $f(x)$ as (10) [18].

\begin{align*}&\hspace {-.5pc} f\left ({x,\gamma }\right)\cong f\left ({0 }\right)+\sum \limits _{k=1}^{l} \sum \limits _{n=1}^\infty a_{kn} C_{kn}(x,\gamma) \\&\qquad\qquad\qquad\qquad\displaystyle { +\sum \limits _{k=1}^{l} \sum \limits _{n=1}^\infty b_{kn} D_{kn}(x,\gamma) } \tag{10}\end{align*}

where,

\begin{align*} C_{kn}(x,\gamma)\triangleq&\frac {\Gamma \left ({n+1 }\right)}{\Gamma \left ({n+\gamma }\right)}\left ({\text {x-k} }\right)^{n+\gamma -1} \\[3pt] D_{kn}(x,\gamma)\triangleq&\frac {\Gamma \left ({n+1 }\right)}{\Gamma \left ({n+\gamma -1 }\right)}{(x-k)}^{n+\gamma -2}\end{align*}

The infinite summation of polynomials is approximated as a finite summation given in (11).

\begin{align*}&\hspace {-.5pc} f\left ({x,\gamma }\right)\cong f\left ({0 }\right)+\sum \limits _{k=1}^{l} \sum \limits _{n=1}^{M} a_{kn} C_{kn}(x,\gamma) \\&\qquad\qquad\qquad\qquad\displaystyle { +\sum \limits _{k=1}^{l} \sum \limits _{n=1}^{M} b_{kn} D_{kn}(x,\gamma) } \tag{11}\end{align*}

Note that, $f\left ({0 }\right),{a}_{kn}$, and $b_{kn}$ are unknown coefficients that need to be found. For the Laplace Transform ($\mathcal {L}$), the following properties are utilized to find (9) [18].

\begin{align*} \mathcal {L}[\left ({x-k }\right)^{n-1}]=&\frac {\mathrm {\Gamma } (n)}{s^{n}}e^{-ks} \text {and } \\ \mathcal {L}\left [{ \frac {d^{\gamma }f\left ({x }\right)}{dx^{\gamma }} }\right]=&s^{\gamma }F\left ({s }\right)-s^{\gamma -1}f\mathrm {(0)} \text {for } 0 < \gamma < 1.\end{align*}

where $\mathcal {L}$ stands for the Laplace transform and $\mathcal {L}\left [{ f\left ({x }\right) }\right]=F\left ({s }\right)$.

To reduce the error between the proposed function $f(x)$ obtained by DAM and real data, the Least Square Method is employed. The unknown coefficients $a_{kn},b_{kn}$, and $f\left ({0 }\right)$ and parameters such as $M,l,\gamma $, are optimized by minimizing the squared total error. The squared total error $\epsilon _{T}^{2}$ is defined as the error between the approximated function $f(x)$ and the actual data and shown in (12):

\begin{equation*} \epsilon _{T}^{2}=\sum \limits _{i=l}^{m_{1}} \left ({P_{i}-f\left ({i,\gamma }\right) }\right)^{2}\tag{12}\end{equation*}

The error $\epsilon _{T}^{2}$ is minimized by a gradient-based approach as the following.

\begin{align*} \frac {\partial \epsilon _{T}^{2}}{\partial f(0)}=&0, \\ \frac {\partial \epsilon _{T}^{2}}{\partial a_{rt}}=&0,\end{align*}

and

\begin{equation*} \frac {\partial \epsilon _{T}^{2}}{\partial b_{rt}}=0.\end{equation*}

where, $r=1,2,3,\ldots l$ and $t=1,2,3,\ldots M$.

Then, a system of linear algebraic equations (SLAE) given in (13) is created where $\left [{ \boldsymbol {B} }\right]$ the matrix contains all unknowns ${a}_{kn}$, $b_{kn}$ and $f\left ({0 }\right)$.

\begin{equation*} \left [{ \boldsymbol {A} }\right]\left [{ \boldsymbol {B} }\right]=\left [{ \boldsymbol {C} }\right]\tag{13}\end{equation*}

[$\boldsymbol {A}$], [$\boldsymbol {B}$], and [$\boldsymbol {C}$] are shown in (14), (15), and (16), as shown at the bottom of the next page, respectively. Optimum values of $M,l,\gamma $ are found by grid search. As mentioned above, $\gamma $ is constrained in (0, 1) region.

\begin{align*} \boldsymbol {A}=\left [{ {\begin{array}{cccccccccccccccccccc} \boldsymbol {A}_{ \boldsymbol {1,1}} &\quad \boldsymbol {A}_{ \boldsymbol {1,2}}\\ \boldsymbol {A}_{ \boldsymbol {2,1}} &\quad \boldsymbol {A}_{ \boldsymbol {2,2}}\\ \end{array}} }\right]\tag{14}\end{align*}

**Figure:** \begin{align*} \boldsymbol {A}_{ \boldsymbol {1,1}}=&\left [{\! {\begin{array}{cccccccccccccccccccc} m_{1}\!-\!l\!+\!1 &~\, \displaystyle \sum \limits _{i=l}^{m_{1}} C_{11} &~\, \ldots &~\, \displaystyle \sum \limits _{i=l}^{m_{1}} C_{1M} \\[8pt] \displaystyle \sum C_{11} &~\, \displaystyle \sum \limits _{i=l}^{m_{1}} {C_{11}C_{11}} &~\, \ldots &~\, \displaystyle \sum \limits _{i=l}^{m_{1}} {C_{1M}C_{11}} \\[8pt] \displaystyle \sum C_{12} &~\, \displaystyle \sum \limits _{i=l}^{m_{1}} {C_{11}C_{12}} &~\, \ldots &~\, \displaystyle \sum \limits _{i=l}^{m_{1}} {C_{1M}C_{12}} \\ \vdots &~\, \vdots &~\, \vdots &~\, \vdots \\ \displaystyle \sum C_{lm} &~\, \displaystyle \sum \limits _{i=l}^{m_{1}} {C_{11}C_{lM}} &~\, \ldots &~\, \displaystyle \sum \limits _{i=l}^{m_{1}} {C_{1M}c_{lM}} \\[8pt] \end{array}}{\begin{array}{cccccccccccccccccccc} \displaystyle \sum \limits _{i=l}^{m_{1} }C_{21} &~\, \ldots &~\, \displaystyle \sum \limits _{i=l}^{m_{1}} C_{2M} \\[8pt] \displaystyle \sum \limits _{i=l}^{m_{1}} {C_{21}C_{11}} &~\, \ldots &~\, \displaystyle \sum \limits _{i=l}^{m_{1}} {C_{2M}C_{11}} \\[8pt] \displaystyle \sum \limits _{i=l}^{m_{1}} {C_{21}C_{12}} &~\, \ldots &~\, \displaystyle \sum \limits _{i=l}^{m_{1}} {C_{2M}C_{12}} \\ \vdots &~\, \vdots &~\, \vdots \\ \displaystyle \sum \limits _{i=l}^{m_{1}} {C_{21}C_{lM}} &~\, \ldots &~\, \displaystyle \sum \limits _{i=l}^{m_{1}} {C_{2M}C_{lM}} \\[8pt] \end{array}}{\begin{array}{cccccccccccccccccccc} \ldots &~\, \displaystyle \sum \limits _{i=l}^{m_{1}} C_{l1} &~\, \ldots &~\, \displaystyle \sum \limits _{i=l}^{m_{1}} C_{lM} \\[8pt] \ldots &~\, \displaystyle \sum \limits _{i=l}^{m_{1}} {C_{l1}C_{11}} &~\, \ldots &~\, \displaystyle \sum \limits _{i=l}^{m_{1}} {C_{lM}C_{11}} \\[8pt] \ldots &~\, \displaystyle \sum \limits _{i=l}^{m_{1}} {C_{l1}C_{12}} &~\, \ldots &~\, \displaystyle \sum \limits _{i=l}^{m_{1}} {C_{lM}C_{12}} \\ \vdots &~\, \vdots &~\, \vdots &~\, \vdots \\ \ldots &~\, \displaystyle \sum \limits _{i=l}^{m_{1}} {C_{l1}C_{lM}} &~\, \ldots &~\, \displaystyle \sum \limits _{i=l}^{m_{1}} {C_{lM}C_{lM}} \\ \end{array}} \!}\right] \\ \boldsymbol {A}_{ \boldsymbol {2,1}}=&\left [{\! {\begin{array}{cccccccccccccccccccc} \displaystyle \sum D_{11} &~\, \displaystyle \sum \limits _{i=l}^{m_{1}} {C_{11}D_{11}} &~\, \ldots &~\, \displaystyle \sum \limits _{i=l}^{m_{1}} {C_{1M}D_{11}} \\[8pt] \displaystyle \sum \limits _{i=l}^{m_{1}} D_{12} &~\, \displaystyle \sum \limits _{i=l}^{m_{1}} {C_{11}D_{12}} &~\, \ldots &~\, \displaystyle \sum \limits _{i=l}^{m_{1}} {C_{1M}D_{12}} \\ \vdots &~\, \vdots &~\, \vdots &~\, \vdots \\ \displaystyle \sum \limits _{i=l}^{m_{1}} D_{lM} &~\, \displaystyle \sum \limits _{i=l}^{m_{1}} {C_{11}D_{lM}} &~\, \ldots &~\, \displaystyle \sum \limits _{i=l}^{m_{1}} {C_{1M}D_{lM}} \\ \end{array}}{\begin{array}{cccccccccccccccccccc} \displaystyle \sum \limits _{i=l}^{m_{1}} {C_{21}D_{11}} &~\, \ldots &~\, \displaystyle \sum \limits _{i=l}^{m_{1}} {C_{2M}D_{11}} \\[8pt] \displaystyle \sum \limits _{i=l}^{m_{1}} {C_{21}D_{12}} &~\, \ldots &~\, \displaystyle \sum \limits _{i=l}^{m_{1}} {C_{2M}D_{12}} \\ \vdots &~\, \vdots &~\, \vdots \\ \displaystyle \sum \limits _{i=l}^{m_{1}} {C_{21}D_{lM}} &~\, \ldots &~\, \displaystyle \sum \limits _{i=l}^{m_{1}} {C_{2M}D_{lM}} \\ \end{array}}{\begin{array}{cccccccccccccccccccc} \ldots &~\, \displaystyle \sum \limits _{i=l}^{m_{1}} {C_{l1}D_{11}} &~\, \ldots &~\, \displaystyle \sum \limits _{i=l}^{m_{1}} {C_{lM}D_{11}} \\[8pt] \ldots &~\, \displaystyle \sum \limits _{i=l}^{m_{1}} {C_{l1}D_{12}} &~\, \ldots &~\, \displaystyle \sum \limits _{i=l}^{m_{1}} {C_{lM}D_{12}} \\ \vdots &~\, \vdots &~\, \vdots &~\, \vdots \\ \ldots &~\, \displaystyle \sum \limits _{i=l}^{m_{1}} {C_{l1}D_{lM}} &~\, \ldots &~\, \displaystyle \sum \limits _{i=l}^{m_{1}} {C_{lM}D_{lM}} \\ \end{array}} \!}\right] \end{align*}

\begin{align*}\boldsymbol {A}_{ \boldsymbol {1,2}}=&\left [{ {\begin{array}{cccccccccccccccccccc} \displaystyle \sum \limits _{i=l}^{m_{1}} D_{11} &\quad \ldots &\quad \displaystyle \sum \limits _{i=l}^{m_{1}} D_{1M} &\quad \ldots &\quad \displaystyle \sum \limits _{i=l}^{m_{1}} D_{lM} \\[8pt] \displaystyle \sum \limits _{i=l}^{m_{1}} {D_{11}C_{11}} &\quad \ldots &\quad \displaystyle \sum \limits _{i=l}^{m_{1}} {D_{1M}C_{11}} &\quad \ldots &\quad \displaystyle \sum \limits _{i=l}^{m_{1}} {D_{lM}C_{11}} \\[8pt] \displaystyle \sum \limits _{i=l}^{m_{1}} {D_{11}C_{12}} &\quad \ldots &\quad \displaystyle \sum \limits _{i=l}^{m_{1}} {D_{1M}C_{12}} &\quad \ldots &\quad \displaystyle \sum \limits _{i=l}^{m_{1}} D_{lM} C_{12} \\ \vdots &\quad \vdots &\quad \vdots &\quad \vdots &\quad \vdots \\ \displaystyle \sum \limits _{i=l}^{m_{1}} {D_{11}C_{lM}} &\quad \ldots &\quad \displaystyle \sum \limits _{i=l}^{m_{1}} {D_{1M}C_{lM}} &\quad \ldots &\quad \displaystyle \sum \limits _{i=l}^{m_{1}} {D_{lM}C_{lM}} \\ \end{array}} }\right] \\ \boldsymbol {A}_{ \boldsymbol {2,2}}=&\left [{ {\begin{array}{cccccccccccccccccccc} \displaystyle \sum \limits _{i=l}^{m_{1}} {D_{11}D_{11}} &\quad \ldots &\quad \displaystyle \sum \limits _{i=l}^{m_{1}} {D_{1M}D_{11}} & \quad \ldots &\quad \displaystyle \sum \limits _{i=l}^{m_{1}} D_{lM} D_{11}\\[8pt] \displaystyle \sum \limits _{i=l}^{m_{1}} {D_{11}D_{12}} & \quad \ldots &\quad \displaystyle \sum \limits _{i=l}^{m_{1}} {D_{1M}D_{12}}&\quad \ldots &\quad \displaystyle \sum \limits _{i=l}^{m_{1}} {D_{lM}D_{12}}\\ \vdots &\quad \vdots &\quad \vdots &\quad \vdots &\quad \vdots \\ \displaystyle \sum \limits _{i=l}^{m_{1}} {D_{11}D_{lM}} & \ldots & \displaystyle \sum \limits _{i=l}^{m_{1}} {D_{1M}D_{lM}} & \ldots & \displaystyle \sum \limits _{i=l}^{m_{1}} {D_{lM}D_{lM}} \\ \end{array}} }\right] \\ \left [{ \boldsymbol {B} }\right]=&{\begin{array}{cccccccccccccccccccc} \big[f\left ({0 }\right) &~a_{11} &~a_{12} &~\ldots &~a_{1M}\\ \end{array}}{\begin{array}{cccccccccccccccccccc} a_{21} &~a_{22} &~\ldots \\ \end{array}}~{\begin{array}{cccccccccccccccccccc} a_{2M} &~\ldots ~a_{l1}\ldots &~a_{lM}\\ \end{array}} {\begin{array}{cccccccccccccccccccc} {b}_{11} &~b_{12}\\ \end{array}}{\begin{array}{cccccccccccccccccccc} b_{1M} &~b_{21} &~\ldots \\ \end{array}}~{\begin{array}{cccccccccccccccccccc} {b}_{2M} &~\ldots &~b_{l1}\\ \end{array}}{\begin{array}{cccccccccccccccccccc} b_{l2} &~\ldots &~b_{lM}\\ \end{array}}\big]^{T} \\ \tag{15}\\{}\big[\boldsymbol {C}\big]=&\left [{ {\begin{array}{cccccccccccccccccccc} \displaystyle \sum \limits _{i={l}}^{m_{1}} {P}_{i} &\quad \displaystyle \sum \limits _{i={l}}^{m_{1}} {P_{i}{C}_{11}} &\quad \displaystyle \sum \limits _{i={l}}^{m_{1}} {P_{i}{C}_{12}} &\quad {\ldots } &\quad \displaystyle \sum \limits _{i={l}}^{m_{1}} {P_{i}{C}_{lM}} &\quad \displaystyle \sum \limits _{i={l}}^{m_{1}} {P_{i}{D}_{11}} &\quad \displaystyle \sum \limits _{i={l}}^{m_{1}} {P_{i}{D}_{12}} {\ldots } &\quad \displaystyle \sum \limits _{i={l}}^{m_{1}} {P_{i}{D}_{lM}} \\ \end{array}} }\right]^{T}\tag{16}\end{align*}

${A}$ matrix consists of the four main blocks below. Note that, in the matrix, the abbreviations are used as $C_{kn}\left ({x,\gamma }\right)=C_{kn}$ and $D_{kn}\left ({x,\gamma }\right)=D_{kn}$.

**FIGURE 1.**
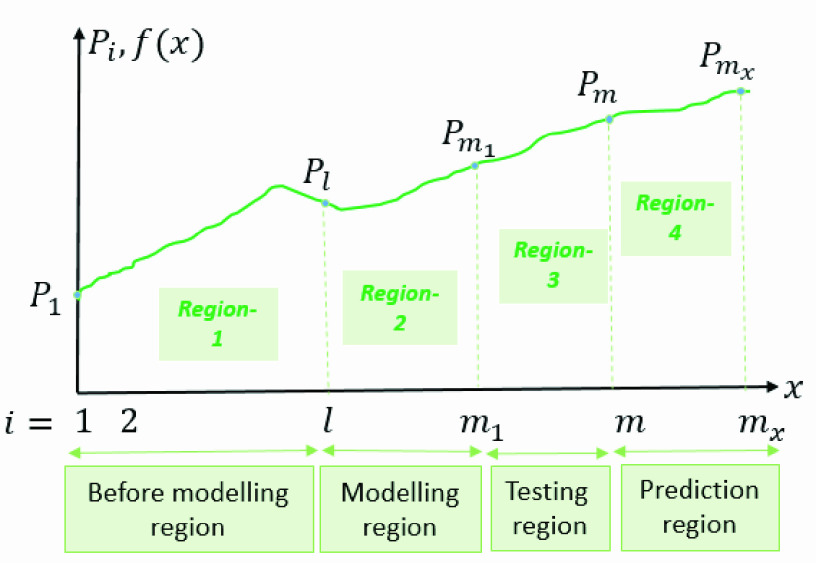
The regions of the dataset [3].

With a data sequence vector ${ \boldsymbol {P}= [P}_{1},P_{2},\ldots,P_{m}$] that contains $m$ points, DAM divides the range of the data into 4 regions in total including the prediction range. This is shown in Fig. 1 where the horizontal axis represents time. $P_{i}$ denotes the data for $i^{th}$ date where $i < m$. Since the beginning of infection is different for each country, the length of the dataset used for modeling varies. For example, $P_{2}$ is the COVID-19 infected case numbers of a country in the second recorded day of their dataset. The region division is required because of parameters given in the previous section (10) such as $M,l,\gamma $ need to be found separately for modeling and prediction. Here the first three regions consist of the actual data where region 4 contains unknown future values predicted by the method itself. DAM models any time instant in terms of previous $l$ values, therefore the first $l$ value is taken as “historical data”. The second region is the “modeling region”. Then, Region 3 comes as the “Testing Region” which is used to understand the performance of modeling. Finally, Region 4 is the “prediction region” where the aim is to predict the next upcoming data, in our case, COVID-19 infected case numbers.

For modeling Region 2, the optimum values of coefficients $a_{kn},b_{kn},M, l,\gamma $, and $f\left ({0 }\right)$ are determined by employing Least Squares Method.

After modeling the Region 2, the performance of the method is measured with Region 3. DAM makes a one-step prediction at a time because every time instant $f\left ({x_{i} }\right)$ depends on anterior values including $f\left ({x_{i-1} }\right)$. When there are no actual previous data for calculating a particular time instant, previously predicted values are used. Otherwise, parameters are updated in an online fashion according to the data at hand. With the help of function $f(x)$, the first value of the testing region, $f(m_{1})$ of Figure 1, is calculated. Here $f(m_{1})$ corresponds to the modeling of actual data $P_{m_{1}}$. Then, since the testing region has actual data, point x $= m_{1}$ and its corresponding value $P_{m_{1}}$ are included in the modeling region and $f\left ({0 }\right),a_{kn},b_{kn},M,\mathrm { }l,\gamma $ values are adjusted before the next point is calculated (i.e. $m_{1}+1$, $f(m_{1}+1))$. After value $f\left ({m_{1}+1 }\right)$ was obtained from modeling, the value is stored and the same procedure is followed to find $(f\left ({m_{1}+2 }\right))$. These operations are repeated until the last value of the testing region, is calculated. In our experiments, $m=April\,\,19$.

The last region is called the “Prediction Region” where there are no actual data. In this region, the first prediction $f\left ({m+1 }\right)$ is found by using the coefficients and unknowns obtained by the tuning approach in the testing region. After that, the first predicted value ($f\left ({m+1 }\right))$ is included in Region 3 (testing) for the consecutive prediction $f\left ({m+2 }\right)$. This procedure is iterative and continues up to $f\left ({m_{x} }\right)$.

**FIGURE 2.**
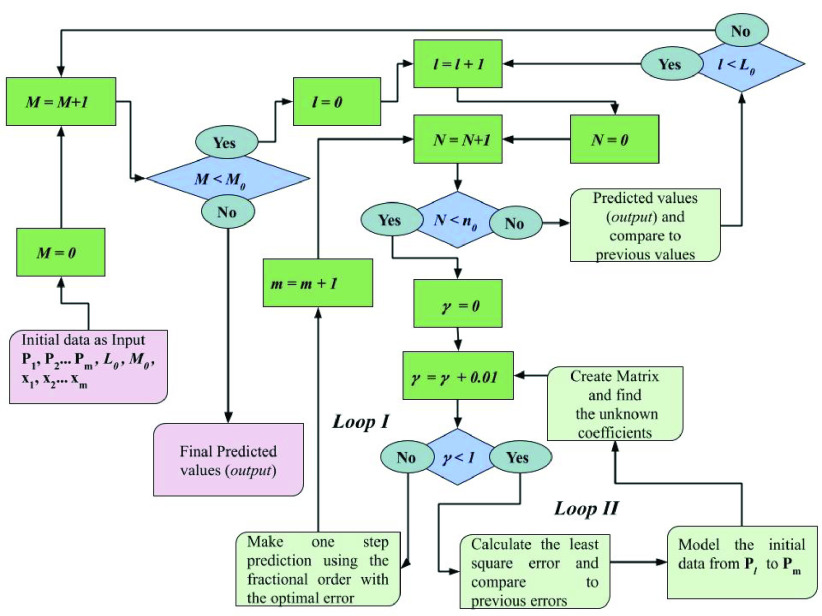
The algorithm for the prediction [3].

The algorithm for the prediction with DAM is given in Fig. 2. The first step is initializing the parameters ($l,\mathrm { }M{,x}_{1},x_{2},\ldots x_{m}$ and ${P}_{1},P_{2},\mathrm { }\ldots P_{m},L_{0}$ and $M_{0}$). Here, $L_{0}$ and $M_{0}$ are predefined values and determine how many steps should be taken in the optimization of $l$ and $M$ values. Then, the counter variable $N$ is introduced, which sets the number of prediction steps.

The total number of prediction steps is given as $n_{0}$. The fractional-order $\gamma $ starts from 0 to 1 with the increment is 0.01 for each loop trying to find the optimized value. Note that, $\gamma = 1$ corresponds to the integer-order derivative and it is included in the search region of optimization. For each value of $\gamma $ between 0 and 1, matrix ${A}$ given as (14) is formed, and then, the unknown constant coefficients are given in (10) are evaluated together with the parameters $l$ and $M$. After modeling, for each testing data, the value of the error is analyzed and compared to previously obtained values. If it is smaller than the previous one, the corresponding fractional-order value is memorized. At the end of Loop II, the optimal value of the fractional-order, which corresponds to the optimal modeling in the test region is obtained and the coefficients given in (10) are found. Subsequently, the prediction for the next upcoming value is made with the same equation. Afterward, all the iterative procedures starting from the increment for N is repeated so that the previously predicted value is included in the initial data for the next step prediction. This process continues up to the end of Loop I. Finally, $n_{0}$ the number of predictions is obtained. Keep in mind that, for the parameters l and M, there exist two loops starting from 1 to $l_{0}$ and 1 to $M_{0}$ trying to find the optimum values of the coefficients to get the outcomes with a minimum error for the testing region 4, 8.

DAM is a general framework and can be applied to many time series modeling problems. Here we apply it to model and predict the ongoing pandemic, COVID-19. In this study, the COVID-19 case numbers of countries are modeled until the 19^th^ of April, 2020. The date 19^th^ of April is in Region 3 as testing to predict for next values.

### B. Time-Dependent Sir Modelling and Prediction Approach

We employ the Time-dependent susceptible-infected-recove- red model (SIR) that is described in detail by Lu *et al.* [21] to compare it with DAM. It is a common and well-known pandemic model, upgraded by using variables as functions of time. From ordinary differential equations of the traditional SIR model, with most recent data of that day, we have with $n$ (total population):

\begin{align*} S\left ({t+1 }\right)-S\left ({t }\right)=&\frac {-\beta \left ({t }\right)S\left ({t }\right)I(t)}{n} \tag{17}\\ I\left ({t+1 }\right)-I\left ({t }\right)=&\frac {\beta \left ({t }\right)S\left ({t }\right)I\left ({t }\right)}{n}-\gamma \left ({t }\right)I(t)\quad \tag{18}\\ R\left ({t+1 }\right)-R\left ({t }\right)=&\gamma \left ({t }\right)I(t)\tag{19}\end{align*}

The formulas for $\beta (t)$ and $\gamma (t)$ are derived from the above equations and the followings are obtained:

\begin{align*} \gamma \left ({t+1 }\right)=&\frac {R\left ({t+1 }\right)-R\left ({t }\right)}{I(t)}\tag{20}\\ \beta \left ({t+1 }\right)=&\frac {n[I\left ({t+1 }\right)-I\left ({t }\right)+R\left ({t+1 }\right)-R\left ({t }\right)] }{I\left ({t }\right)[n-I\left ({t }\right)-R(t)] }\tag{21}\end{align*}

The approach is using Finite Impulse Response (FIR) and Ridge Regression to track and predict the transmission rate and recovering rate based on historical numbers with elements of machine learning algorithms. This tracking and the predicting process is programmed on Python, see more in [21]. In general, $\beta (t)$ and $\gamma (t)$ at time $t$ are predicted based on previous data of themselves as below:

\begin{align*} \beta \left ({t }\right)=&\sum \limits _{j=1}^{J} {a_{j}\beta \left ({t-j }\right)+a_{0}} \tag{22}\\ \gamma \left ({t }\right)=&\sum \limits _{k=1}^{K} {b_{k}\gamma \left ({t-k }\right)+b_{0}}\tag{23}\end{align*}

where $J$ and $K$ are the orders of the two FIR filters, $a_{j}$ and $b_{k}$ are the coefficients of the impulse responses of these two FIR filters. These coefficients $a_{j}$ and $b_{k}$ are estimated by the Ridge Regression method:

\begin{align*}&\min _{a_{j}}{\sum \limits _{t=J}^{T-2} \left ({\beta \left ({t }\right)-\hat {\beta }(t) }\right)^{2} +\alpha _{1}\sum \limits _{j=0}^{J} a_{j}^{2}} \tag{24}\\&\min _{b_{k}}{\sum \limits _{t=K}^{T-2} \left ({\gamma \left ({t }\right)-\hat {\gamma }(t) }\right)^{2} +\alpha _{2}\sum \limits _{k=0}^{K} b_{k}^{2}}\tag{25}\end{align*}

where $\alpha _{1}$ and $\alpha _{2}$ are the regularization parameters, $\hat {\beta }(t)$ and $\hat {\gamma }(t)$ are the predicted transmission rate and recovering rate. After predicting the value for $\beta (t)$ and $\gamma (t)$, we substitute them into (18) and (19) to find out the estimated number of infected and recovered cases of the next days.

### C. Modeling and Prediction of the Peak with Gaussian Distribution

This section briefly explains how to model and predict the peak time and value for the daily confirmed COVID-19 cases using DAM. After modeling the discrete finite data, the prediction of the peak value for the total confirmed cases can be found by assuming that the derivative of the total confirmed cases has Gaussian distribution. The number of daily confirmed cases follows an increasing trend in the first period of the pandemic and it is expected that this number decreases by time with precautions and the immune system. Therefore, it is reasonable to assume the number of daily confirmed cases curve has a bell shape. This way, the future of total confirmed cases and the peak time of the infection can be modeled and predicted. Using this approach, one can model the discrete data at the early stages of the pandemic and find an analytical expression that represents the dataset with minimum error.

In our case, the dataset which is modeled with DAM has the analytical expression $f\left ({x }\right)$ as given in (10) for the total confirmed data in a specific interval. Since the discrete dataset now has a continuous form, the change of the total confirmed case over time is represented as $g(x)$:

\begin{equation*} g\left ({x }\right)=\frac {df(x)}{dx}\tag{26}\end{equation*}

where, $g\left ({x }\right)$ is assumed to be a Gaussian distribution $g\left ({x }\right)=Ae^{-a\left ({\text {x-b} }\right)^{2}}$ with unknown constant coefficients $A$, $a$, and $b$. Here, $A$, $a$, and $b$ are related to the peak of the Gaussian, the variance, and the mean, respectively. For our discrete data, $G_{i}$ is defined as:

\begin{equation*} G_{i}=\left.{ \frac {df\left ({x }\right)}{dx} }\right |_{x=x_{i}}\tag{27}\end{equation*}

Then, from (11), $G_{i}$ is obtained as (28).

\begin{align*} G_{i}=\left [{ \sum \limits _{k} \sum \limits _{n} {a_{kn}\frac {d}{dx}C_{kn}\left ({x }\right)+\sum \limits _{k} \sum \limits _{n} {b_{kn}\frac {d}{dx}D_{kn}\left ({x }\right)}} }\right]_{x=x_{i}} \\\tag{28}\end{align*}

The final expression for $G_{i}$ becomes (29):

\begin{align*}&\hspace {-.5pc} G_{i}=\sum \limits _{k=1}^{l} \sum \limits _{n=1}^{M} {a_{kn}\frac {\Gamma \left ({n+1 }\right)}{\mathrm {\Gamma }\left ({n+\gamma -1 }\right)}} \left ({x_{i}-k }\right)^{n+\gamma -2} \\&\qquad\qquad\qquad\qquad\displaystyle { +\sum \limits _{k=1}^{l} \sum \limits _{n=1}^{M} {b_{kn}\frac {\Gamma \left ({n+1 }\right)(n+\gamma \mathrm {-2)}}{\mathrm {\Gamma }\left ({n+\gamma -1 }\right)}} \left ({x_{i}-k }\right)^{n+\gamma -3} } \\\tag{29}\end{align*}

The same procedure implemented in DAM for optimization, the Least Squares Method is employed where $\epsilon _{T}^{2}$ corresponds to a total error between the proposed Gaussian distribution $g(x)$ and the derivative of the curve representing actual data found by DAM and is as given in (30).

\begin{equation*} \epsilon _{T}^{2}=\sum \limits _{i=0}^{m} \left [{ G_{i}-g(x_{i}) }\right]^{2} \mathrm { =}\sum \limits _{i=0}^{m} \left [{ G_{i}-Ae^{-a\left ({x_{i}-b }\right)^{2}} }\right]^{2}\tag{30}\end{equation*}

A gradient-based error minimization approach is followed to find constant coefficients $A,a$, and $b$.

\begin{equation*} \frac {\partial \epsilon _{T}^{2}}{\partial A}=\sum \limits _{i=0}^{m} {\left [{ G_{i}-Ae^{-a\left ({x_{i}-b }\right)^{2}} }\right]e^{-a\left ({x_{i}-b }\right)^{2}}=0}\tag{31}\end{equation*}

By distributing the exponential term over the terms within the brackets and substituting $B_{i}=e^{-a\left ({x_{i}-b }\right)^{2}}$, (32) is obtained.

\begin{equation*} \sum \limits _{i=0}^{m} {{G_{i}B}_{i}=A} \sum \limits _{i=0}^{m} B_{i}^{2} \mathrm {\to }A=\frac {\sum _{i=0}^{m} {G_{i}B}_{i} }{\sum _{i=0}^{m} B_{i}^{2}}\tag{32}\end{equation*}

After obtaining (32), $\frac {\partial \epsilon _{T}^{2}}{\partial a}=0$ is introduced as (33),

\begin{equation*} \frac {\partial \epsilon _{T}^{2}}{\partial a}=\sum \limits _{i=0}^{m} {\left [{ G_{i}-AB_{i} }\right]B_{i}\left ({x_{i}-b }\right)^{2}=0}\tag{33}\end{equation*}

The final expression for (33) is as follows:

\begin{equation*} \sum \limits _{i=0}^{m} {\left [{ G_{i}B_{i} }\right]\left ({x_{i}-b }\right)^{2}-A\sum \limits _{i=0}^{m} {B_{i}^{2}\left ({x_{i}-b }\right)^{2}=0}}\tag{34}\end{equation*}

After applying the same procedure to $\frac {\partial \epsilon _{T}^{2}}{\partial b}=0$, we get (35):

\begin{equation*} \frac {\partial \epsilon _{T}^{2}}{\partial b}=\sum \limits _{i=0}^{m} {\left [{ G_{i}-AB_{i} }\right]B_{i}\left ({x_{i}-b }\right)=0}\tag{35}\end{equation*}

Then,

\begin{equation*} \sum \limits _{i=0}^{m} {\left [{ G_{i}B_{i} }\right]\left ({x_{i}-b }\right)-A} \sum \limits _{i=0}^{m} {B_{i}^{2}\left ({x_{i}-b }\right)=0}\tag{36}\end{equation*}

We have 3 equations as (32), (34), and (36) with three unknowns. Therefore, unknown coefficients can be found optimally using the Least Square Method.

After obtaining the unknown coefficients, optimal $g\left ({x }\right)$ can be found. Note that, $g(x)$ is the derivative of the $f(x)$ in the data region. The integration of $g(x)$ gives the prediction related to peak value and time of the total confirmed cases.

**FIGURE 3.**
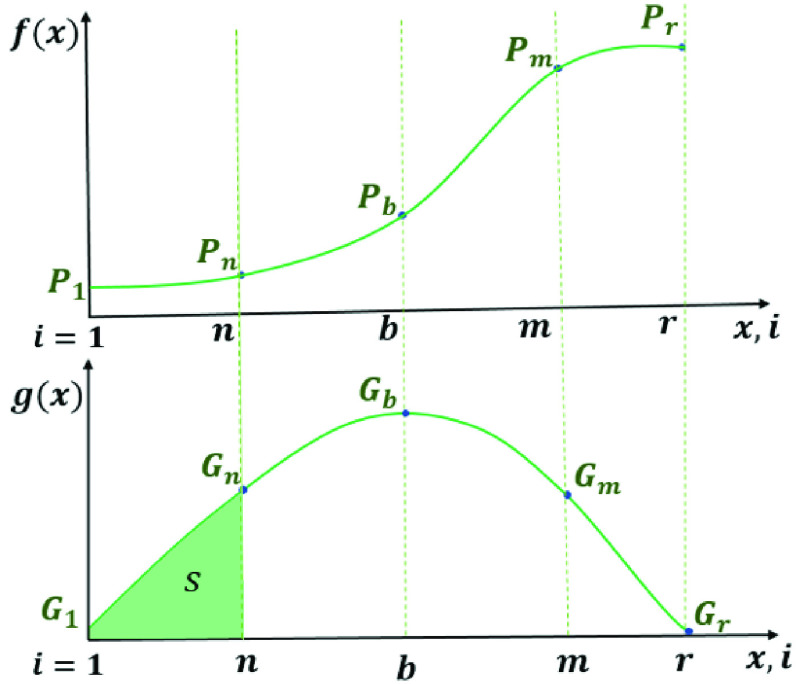
The algorithm for the peak prediction.

In Fig. 3, $f\left ({x }\right)$ and $g(x)$ are illustrated. Here, the actual dataset that contains $m$ values, the finite dataset from $P_{1}$ to $P_{m}$, is shown. Under the assumption of the derivative of $f\left ({x }\right)$ is equal $g(x)$, we can predict for the upcoming values of the function $f\left ({x }\right)$ by employing the Least square method. Note that, to find $f\left ({x }\right)$ for $x>m$, optimum $g(x)$ should be integrated as in (37).

\begin{equation*} P_{r}=f(r)=P_{m}+A\int \limits _{x=m}^{r} {e^{-a\left ({\text {x-b} }\right)^{2}}dx}\tag{37}\end{equation*}

In Fig. 3, ${P}_{r}$ is the predicted value of total confirmed cases at $r^{th}$ day and ${P}_{m}$ is the known last value of total confirmed cases at $m^{th}$ day. Area of region S is equal to ${P}_{n}$. Keep in mind that, the values $1\le x\le n$ corresponds to the historical data (Region-1) in DAM. Consequently, by integrating the Gaussian function, the prediction for $r^{th}$ day is obtained.

A prediction with Gaussian modeling can be summarized as follows. First, the continuous function, which is obtained through DAM that approximates the real dataset $f(x)$ is used to find the daily changes in confirmed cases. Then, by assuming that the daily changes curve is a Gaussian function, unknown parameters ($a,b$, and $A$) of the Gaussian are obtained by Least Square Method that minimizes $\epsilon _{T}^{2}=\sum \limits _{i=0}^{m} \left [{ G_{i}-g(x_{i}) }\right]^{2} $. Finally, by integrating the area under the fitted Gaussian curve, the prediction is obtained as given in (37).

The Gaussian approach models the derivative of the continuous total confirmed cases which are obtained by DAM modeling. Unlike this approach, the SIR model employs discrete total confirmed cases directly. SIR model predicts upcoming days with modeled function while the proposed approach calculates total cases by integrating the modeled curve.

### D. Prediction with Long Short-Term Memory

Conventional Neural Networks that operate on vectors fail to capture time dependencies on sequential data. Recurrent Neural Networks are a family of models that address this issue and are capable of processing over time series. In this study, to compare the performance of DAM on the COVID-19 dataset, we used a special type of recurrent neural network, Long Short-Term Memory (LSTM), that is widely popular in predicting and modeling the content of time series. An LSTM cell in layer- $\boldsymbol {l}$ and step- $\boldsymbol {t}$ has two kinds of inputs, one from the previous time step ($\boldsymbol {h}_{ \boldsymbol {t}- \boldsymbol {1}}^{ \boldsymbol {l}}$) and another from the previous layer ($\boldsymbol {h}_{ \boldsymbol {t}}^{ \boldsymbol {l}- \boldsymbol {1}}$). Information from previous time steps are stored in cell state $({ \boldsymbol {c}}_{ \boldsymbol {t}}^{ \boldsymbol {l}}$) and updated with gated inputs. There are four gates in an LSTM cell: input, forget, output, and gate. Gate $\boldsymbol {g}$ is a hyperbolic tangent (tanh) and takes values between −1 and 1. All other gates are sigmoid functions and are between 0 and 1. LSTMs optionally inherit information from previous time steps with the help of gates. Gate equations are listed below in Equation (38)–(43). Each gate learns its own set of parameters $\boldsymbol {W}$’s and $\boldsymbol {b}$’s. In equations (42) and (43), $\odot $ is the Hadamard Product.

\begin{align*} \boldsymbol {f}_{ \boldsymbol {t}}=&\sigma \left ({\boldsymbol {W}_{ \boldsymbol {f}}[{ \boldsymbol {h}}_{ \boldsymbol {t}- \boldsymbol {1}}^{ \boldsymbol {l}}, \boldsymbol {h}_{ \boldsymbol {t}}^{ \boldsymbol {l}- \boldsymbol {1}}]+ \boldsymbol {b}_{ \boldsymbol {f}} }\right) \tag{38}\\ \boldsymbol {i}_{ \boldsymbol {t}}=&\sigma \left ({\boldsymbol {W}_{ \boldsymbol {i}}[{ \boldsymbol {h}}_{ \boldsymbol {t}- \boldsymbol {1}}^{ \boldsymbol {l}}, \boldsymbol {h}_{ \boldsymbol {t}}^{ \boldsymbol {l}- \boldsymbol {1}}]+ \boldsymbol {b}_{ \boldsymbol {i}} }\right) \tag{39}\\ \boldsymbol {o}_{ \boldsymbol {t}}=&\sigma \left ({\boldsymbol {W}_{ \boldsymbol {o}}[{ \boldsymbol {h}}_{ \boldsymbol {t}- \boldsymbol {1}}^{ \boldsymbol {l}}, \boldsymbol {h}_{ \boldsymbol {t}}^{ \boldsymbol {l}- \boldsymbol {1}}]+ \boldsymbol {b}_{ \boldsymbol {o}} }\right) \tag{40}\\ \boldsymbol {g}_{ \boldsymbol {t}}=&\tanh \left ({\boldsymbol {W}_{ \boldsymbol {g}}[{ \boldsymbol {h}}_{ \boldsymbol {t}- \boldsymbol {1}}^{ \boldsymbol {l}}, \boldsymbol {h}_{ \boldsymbol {t}}^{ \boldsymbol {l}- \boldsymbol {1}}]+ \boldsymbol {b}_{ \boldsymbol {g}} }\right) \tag{41}\\ \boldsymbol {c}_{ \boldsymbol {t}}^{ \boldsymbol {l}}=&\boldsymbol {f}_{ \boldsymbol {t}}\odot \boldsymbol {c}_{ \boldsymbol {t}- \boldsymbol {1}}^{ \boldsymbol {l}}+ \boldsymbol {i}_{ \boldsymbol {t}}\odot \boldsymbol {g}_{ \boldsymbol {t}} \tag{42}\\ \boldsymbol {h}_{ \boldsymbol {t}}^{ \boldsymbol {l}}=&\boldsymbol {o}_{ \boldsymbol {t}} \odot tanh \left ({\boldsymbol {c}_{ \boldsymbol {t}}^{ \boldsymbol {l}} }\right)\tag{43}\end{align*}

An LSTM unit may consist of one or multiple cells where each cell updates its state with the previous state $\boldsymbol {c}_{ \boldsymbol {t}- \boldsymbol {1}}^{ \boldsymbol {l}}$. Therefore gates, the cell state ($\boldsymbol {c}_{ \boldsymbol {t}}^{ \boldsymbol {l}}$) and hidden state $(\boldsymbol {h}_{ \boldsymbol {t}}^{ \boldsymbol {l}})$ are vectors that contain information from all cells of an LSTM unit. Equation (42) shows how an LSTM cell is updated. Here, **f** gate decides how much of previous knowledge should participate in the current state while $\boldsymbol {i}$ gate decides how much of new input should be acquired. Then LSTM neuron updates its internal hidden state by multiplying output and squashed version of $\boldsymbol {c}_{ \boldsymbol {t}}^{ \boldsymbol {l}}$. An LSTM unit only outputs its hidden state information $\boldsymbol {h}$, cell states are utilized internally. To compare our previously proposed approach to the COVID-19 dataset, we employed a Vanilla LSTM network with two stacked LSTM layers and a linear prediction layer. Each LSTM layer contains 50 units. The model is trained with the Adam Optimizer and the learning rate is set to 0.001 [55].

## III. Numerical Results

In this section, we report modeling, prediction, and incubation period analysis of COVID-19 cases for 8 countries using the proposed approaches in comparison to Time-Dependent SIR and LSTM models. COVID19 dataset used in this study is retrieved from [56] and contains numbers of confirmed, recovered, and death cases. Proposed approaches are implemented on MATLAB and publicly available at [57] and [58]. First, the performance of Deep Assessment Methodology on modeling and prediction are investigated. Later, the peak of COVID-19 infection is predicted using the Gaussian Distribution modeling approach. Further, we compared DAM prediction performance to other prediction approaches, Time-Dependent SIR Model and LSTM. Results are reported using the Mean Average Precision Error (MAPE) metric which is calculated as follows:

\begin{equation*} MAPE=\frac {1}{k}\sum \limits _{i=1}^{k} \left |{ \frac {P\left ({i }\right)-f\left ({i }\right)}{P\left ({i }\right)} }\right | \times 100\tag{44}\end{equation*}

where $k$ is the total number of samples, $P\left ({i }\right)$ is the actual value and $f\left ({i }\right)$ is the predicted value for $i^{\mathrm {th}}$ sample.

### A. Modeling Results with Deep Assessment Methodology

In this part, we illustrate the modeling performance of Deep Assessment on COVID-19 cases.

Modeling results for the number of confirmed cases, deaths, and recoveries using Deep Assessment, are shown in Table 1. With DAM, $l$ value (the number of required previous data) is optimized with grid search and varies across countries. For the modeling of the COVID-19 infected case numbers of each country, the required previous data $l$ of past years used in the algorithm differs after optimization and reported in the second column. Optimized $M$ values can be found in the first column. The Deep Assessment model has a 0.6671%, 0.6957%, and 0.5756% average MAPEs for confirmed cases, deaths, and recoveries, respectively. For confirmed and death cases Turkey is the best-modeled country with the smallest MAPE being 0.13% and 0.0092%, while for recovered cases USA is modeled best with 1.62e-12% MAPE. As mentioned in the previous section, the grid search of the fractional-order parameter includes 1, where it is equal to the integer-order derivative. Column 4 shows that none of the experiments optimized fractional-order as 1. This finding demonstrates the benefit of fractional calculus.

\begin{equation*} {MAPE}_{Modeling}=\frac {100}{m-l+1}\sum \limits _{i=l}^{m} \left |{ \frac {P\left ({i }\right)-f\left ({i,\gamma }\right)}{P\left ({i }\right)} }\right |\tag{45}\end{equation*}

**TABLE 1:** Modeling Results ($\gamma$, $M,l$, and MAPE Values) of 8 Countries Including China, France, Germany, Italy, Turkey, the UK, and the USA for COVID-19 Dataset Using Deep Assessment Methodology

Modeling Results		DAM Results			
		M	${l}$	MAPE	$\boldsymbol {\gamma }$
Confirmed	China	7	20	0.5683	0.18
	France	7	19	1.0726	0.07
	Germany	7	20	0.6013	0.18
	Italy	7	20	0.2756	0.18
	Spain	5	20	0.5941	0.01
	Turkey	6	20	0.1300	0.03
	UK	7	20	1.2367	0.03
	USA	5	20	0.8589	0.2
Death	China	7	20	0.8669	0.39
	France	7	20	2.1636	0.39
	Germany	7	20	0.5136	0.43
	Italy	7	19	0.2783	0.03
	Spain	7	20	0.1541	0.32
	Turkey	7	20	0.0092	0.39
	UK	7	20	0.4310	0.43
	USA	5	20	1.1496	0.11
Recovery	China	7	20	0. 5683	0.18
	France	7	20	0.0004	0.32
	Germany	6	20	0.9463	0.22
	Italy	6	20	0.8944	0.03
	Spain	7	19	0.3488	0.07
	Turkey	7	20	3.04E-10	0.43
	UK	4	20	1.8471	0.39
	USA	7	20	1.61E-12	0.07

**FIGURE 4.**
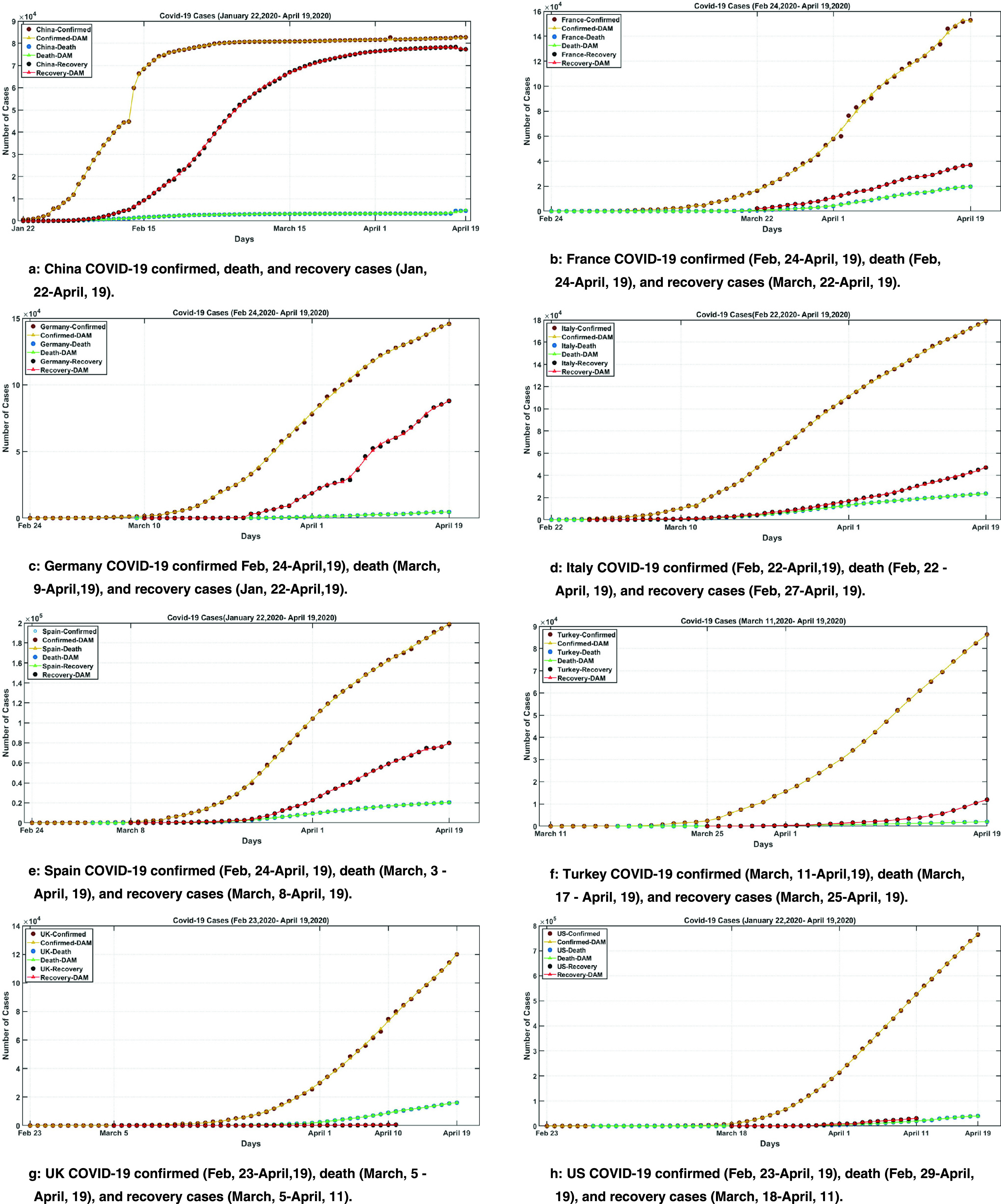
COVID-19 modeling on confirmed, death, and recovered cases for China, France, Italy, Germany, Spain, Turkey, UK, and the USA with DAM Methodology.

Fig. 4 illustrates the modeling performance of each country. As can be seen from the figures, countries with smooth actual data curves like Turkey and Italy result in smaller MAPEs. As expected, jumps in the dataset result in relatively higher MAPEs as in confirmed cases of China and recovered cases of Germany.

### B. One-Step Prediction With DAM and LSTM

This section illustrates the prediction performance of Deep Assessment Methodology on the COVID-19 dataset and compares DAM with LSTM. For all experiments, the test region is set to the 19^th^ of April. Table 2 reports optimized $\gamma $, $l$, $M$ values, and the corresponding performance of both DAM and LSTM models. Here, column 3 reports the performance of DAM while column 6 represents LSTM. Column 3 shows that DAM predicts COVID-19 with an average 0.1343% error while LSTM yields a 0.6393% error. The best-predicted country is Italy for confirmed cases and deaths while France has the smallest MAPE on recoveries. For all three settings, DAM yields the highest MAPE for Spain. Table 2 demonstrates that in the implemented setting, DAM outperforms LSTM by 0.5050% average error and produces fair results.

\begin{equation*} {MAPE}_{Prediction}=\frac {100}{m_{1}-l+1}\sum \limits _{i=m_{1}}^{m} \left |{ \frac {P\left ({i }\right)-f\left ({i,\gamma }\right)}{P\left ({i }\right)} }\right |\tag{46}\end{equation*}

Table 3 reports the prediction of COVID-19 confirmed cases, deaths, and recoveries on April 20th for both DAM and LSTM methods. It can be seen that except for the prediction of recovered cases of Spain, both models produce similar results.

**TABLE 2:** Test ($m_{1} < i < m$) Results ($\gamma$, $l$, $M$, and MAPE) of COVID-19 Confirmed Cases, Deaths, and Recoveries for Corresponding Countries

Test Results		DAM				LSTM	
		M	$\boldsymbol {l}$	MAPE(%) ^*^	$\boldsymbol {\gamma }$	${l}$	MAPE(%)
Confirmed	China	3	20	0.0295	0.39	23	0.0085
	France	6	11	0.0098	0.22	8	0.0157
	Germany	6	5	0.2199	0.11	21	0.0775
	Italy	3	13	0.0089	0.39	11	0.0536
	Spain	4	13	0.2577	0.18	13	0.0916
	Turkey	4	9	0.0435	0.03	11	0.1796
	UK	2	18	0.0801	0.18	24	0.1283
	USA	6	13	0.0209	0.22	14	0.0063
Death	China	3	20	0.0295	0.76	8	7.9000
	France	3	8	0.0196	0.32	10	0.1014
	Germany	6	10	0.0879	0.18	12	0.3231
	Italy	6	15	0.0146	0.07	14	0.1564
	Spain	6	14	0.0531	0.07	8	0.0830
	Turkey	3	7	0.0187	0.03	20	0.000
	UK	3	16	0.0356	0.22	10	3.0697
	USA	4	16	0.0210	0.22	22	0.5126
Recovery	China	4	9	0.0194	0.39	15	0.0865
	France	4	7	0.0065	0.22	7	0.0543
	Germany	5	5	2.0290	0.13	8	0.1124
	Italy	6	6	0.0180	0.07	13	0.1190
	Spain	2	4	0.0603	0.03	12	0.0440
	Turkey	3	12	0.1418	0.32	8	2.2211

^*^ ${MAPE}_{Prediction}$ values

**TABLE 3:** Prediction Results of the Confirmed, Death, and Recovery Cases of the Countries for 20 April 2020

20.04.2020	Model	China	France	Germany	Italy	Spain	Turkey	UK	USA
Confirmed	DAM	83716	155905	148498	181317	199134	90763	125567	789503
	LSTM	82738	156297	147522	181695	200117	89434	120434	788457
	Real	82747	150278	147065	181228	200210	90980	124743	798227
Death	DAM	4901	20036	4402	23956	21007	2145	16920	42223
	LSTM	4057	20915	4752	24098	20820	2140	16370	40783
	Real	4632	20265	4862	24114	20852	2140	16509	42853
Recovery	DAM	77077	37779	90398	49559	82227	12744	–	–
	LSTM	77335	36592	89785	48950	78086	13673	–	–
	Real	77386	37681	91500	48877	81894	13430		

### C. Modelling and Short-Term Prediction With Sir Model

Following Lu *et al.* [21], for the prediction, we set $\alpha _{1}$ and $\alpha _{2}$ to be 0.03 and 10^−6^ respectively for all 8 countries. However, to avoid noising data and due to the trend of each country, we set the orders of the FIR filters $J$ and $K$ as well as choose training data for conditioning and predicting $\beta (t)$ and $\gamma (t)$ differently. The performance, parameters, and settings are reported in Table 4. The first row of the table reports the starting date of training data for prediction. The orders of the FIR filters are illustrated in the second and third rows. The next three rows show MAPE of active cases, recovered cases, and total confirmed cases respectively within training periods for all countries. Note that we exclude data of the 17^th^ of April for China because Wuhan had revised its official death to increase by 1290 deaths. Also, a special note for Spain and U.K is that they stopped updating the recovered number since the 19^th^ of May and 11^th^ of April respectively. Regard to active cases, the lowest MAPE is obtained for the US as 0.64% while China yields the highest value as 3.9945%. In contrast, the lowest MAPE regarding recovered cases is obtained for China as 0.4102% while the US yields the highest value as 1.9007%. However, the MAPE of total confirmed cases, which is the sum of active and recovered number, are just below 0.8% for all countries. Finally, the last three rows report active, recovered and total confirmed cases for each country on date 19^th^ of July, 2020.

**TABLE 4:** MAPE of Training Period (Until 19 June) and Prediction Results of the Countries

Results		China*	France	Germany	Italy	Spain*	Turkey	U.K*	U.S
Starting date		13 Feb	18 Mar	22 Mar	7 Mar	21 Mar	28 Mar	18 Mar	26 Mar
J		3	4	4	5	4	5	4	3
K		3	5	6	5	4	4	4	3
MAPE (%)	Active case	3.9945	1.5090	1.9115	1.0586	1.3998	0.8976	0.8643	0.6400
	Recovered	0.4102	1.2024	1.6285	0.9687	0.9580	1.1190	1.2242	1.9007
	Total confirmed	0.0520	0.7552	0.3022	0.3556	0.3387	0.2359	0.7898	0.2690
19.07.2020									
- Active case		52	56961	4119	7216	71403	11949	276610	1547882
- Recovered case		83475	110090	195951	235225	225315	188886	46680	1683074
- Total confirm		83527	167051	200071	242442	296718	200835	323290	3230956

*China: We exclude data of the 17^th^ of April when Wuhan revised its official death to increase by 1290 deaths.

*Spain: It stopped updating the recovered number since the 19^th^ of May.

*UK: It stopped updating the recovered number since the 11^th^ of April.

**FIGURE 5.**
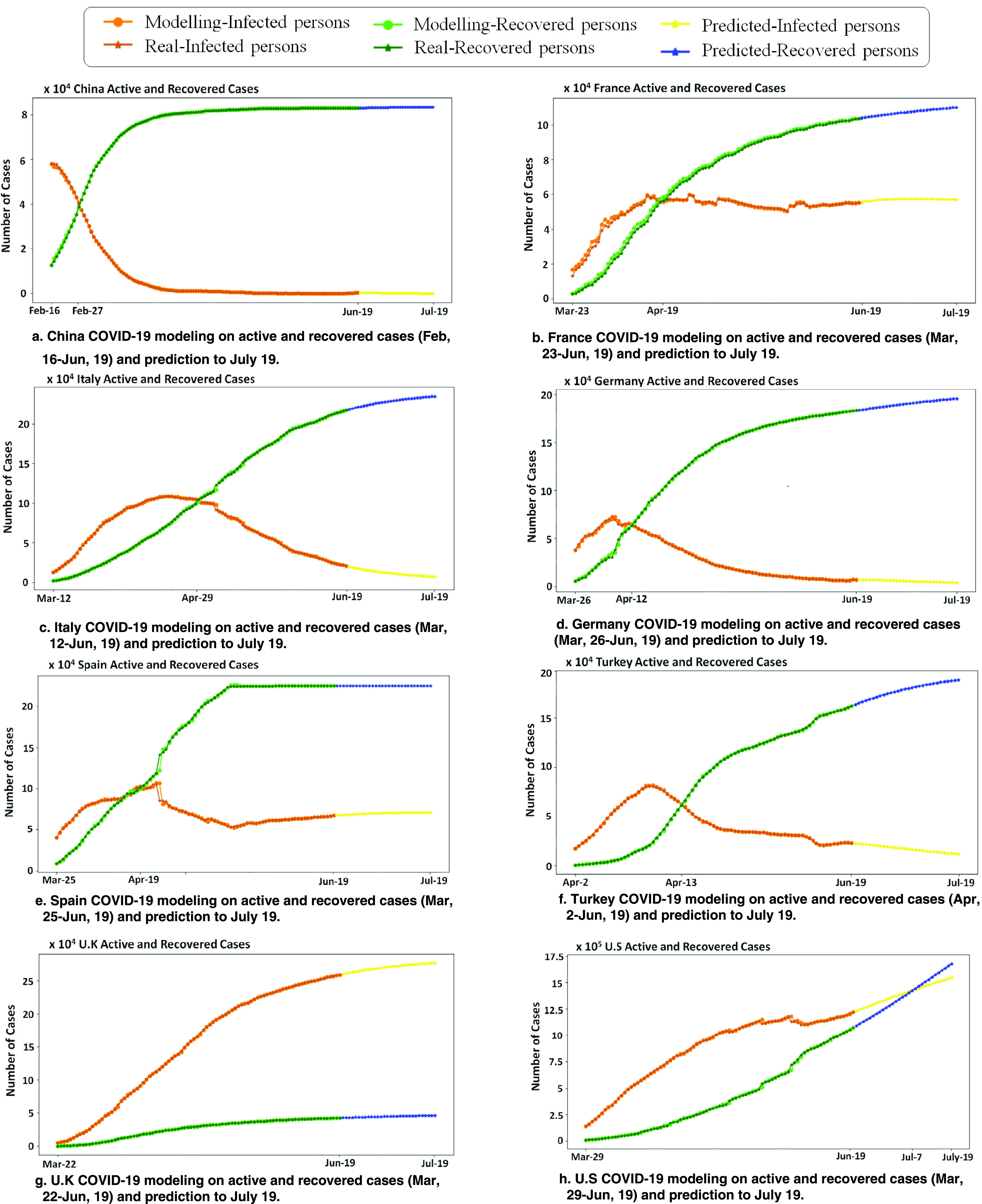
COVID-19 modeling on active and recovered cases for China, France, Italy, Germany, Spain, Turkey, UK, and the USA with SIR Model.

The modeled and predicted active and recovered cases are illustrated in Fig. 5.

### D. Peak and Short-Term Prediction With Gaussian

In this section, the peak of daily confirmed new cases and short-term trends of COVID-19 (30-days ahead) are predicted using Gaussian modeling with DAM. As explained in Section III-C, Deep Assessment Methodology is required for peak estimation. First, the data is modeled using DAM, then the derivative of the modeled data is fitted to a Gaussian by Least Square Method, and relevant parameters such as $a,A$, and $b$ are optimized. Once, the parameters of Gaussian are determined, the future prediction is made based on the model at hand. In this setting, for the modeling with DAM, the total confirmed cases and daily new confirmed cases until the 19^th^ of June are used. The Prediction range is 30 days, from the 19^th^ of June to the 19^th^ of July. In Table 5, the performance, parameters, and settings are reported for Gaussian peak modeling. The first row of the table, reports the Cut value, the number of points eliminated during Gaussian fitting to achieve better optimization because taking the derivative of the edge points in a finite domain causes jumps. For all countries, *M,* number of terms in the equation, is set to 3, as reported in the second row. Fractional order $\gamma $’s are illustrated in the third row. The largest fractional order is obtained for the UK. For Germany and Spain, $\gamma $ is determined equally, being 0.54. Performance of DAM on modeling the data until the 19^th^ of June is shown in the fifth row. The lowest MAPE is obtained for Italy as 0.0229% while Germany yields the highest value as 1.2281%. The average MAPE on modeling is 0.5429%. After taking the derivative of modeled data, Gaussian’s parameters are found by Least Squares Method and the parameter related to variance ($a$), can be seen in row 5. In row 6, $b$ value is reported, which represents the time of peak after the beginning of the infection. For instance, China reached a peak 10 days after the first confirmed case. The latest peak is modeled for the USA as 66. The number of daily changes for the peak date is $A$ in the Gaussian modeling and shown in the seventh row. The highest change is predicted for the USA. Finally, the last row reports total confirmed cases for each country on date 19^th^ of July 2020. The determined parameters $a$ and $b$ of gaussian are on row 5 and 6, respectively. One can see from the 8^th^ column, the latest peak date is observed in the USA and the fastest peak date is obtained for China. It is illustrated that, for modeling, the Gaussian model produces superior results with smaller M value when compared to plain Deep Assessment Methodology. With $M=3$, Gaussian yields smaller MAPE compared to $M=7$ in DAM.

**TABLE 5:** Prediction Results of the Confirmed and Daily Changes in the Countries

Gauss Results	China	France	Germany	Italy	Spain	Turkey	UK	USA
Cut	7	11	9	5	9	4	8	10
M	3	3	3	3	3	3	3	3
$\boldsymbol {\gamma }$	0.01	0.65	0.65	0.75	0.09	0.32	0.33	0.38
MAPE(%)	0.0875	0.0172	1.2281	0.0229	1.1671	0.6667	0.5586	0.5956
$\boldsymbol {a}$	0.004	0.0023	0.0027	0.0012	0.0012	0.0014	0.000927	0.000426
$\boldsymbol {b}$	10	41	38	42	42	37	59	66
$\boldsymbol {A}$	6535.5	3946.4	5179.5	4862.2	6208.9	3833.0	5302.1	30292.6
19.07.2020	83325	159452	190660	238023	245575	185299	304237	2459714

**FIGURE 6.**
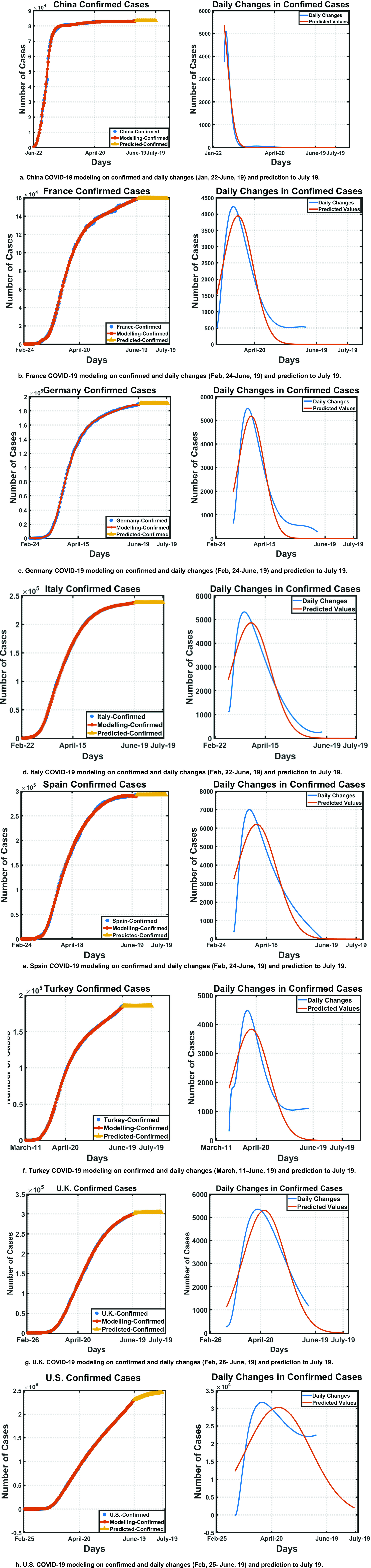
COVID-19 modeling on confirmed and daily changes for China, France, Italy, Germany, Spain, Turkey, UK, and the USA. Left-hand side figures demonstrate total confirmed case numbers, while the right-hand side figures illustrate daily changes in confirmed cases.

The modeled and predicted confirmed cases are illustrated in Fig. 6. The left-hand side figures show the original data, the modeling, and the prediction curves of total confirmed cases. The modeling curve is obtained using DAM, after that, the prediction curve is obtained by fitting the derivative of modeled data to the Gaussian function. Right-hand side figures illustrate the actual data and fitted Gaussians which are the predicted values. Fig. 6 show that peak predictions are reasonably close to real data except for the US. When analyzed together with Table 5, it is seen that Germany yields the highest MAPE due to fluctuations in the actual data. However, the daily change curve of France is the best-fitted one. When compared with the prediction results of the SIR model reported in Table 4, the Gaussian approach underestimates the trend of the pandemic and yields lower values of predictions. The reason for that is two-folded. First, the time-dependent SIR model takes the dynamics of pandemic into accounts such as transmission rate, recovering rate, and the reproduction numbers. On the other hand, Gaussian modeling relies on directly confirmed cases data without using any variant related to pandemic. Secondly, on Gaussian modeling, a Gaussian function is fitted to the daily changes in confirmed cases by optimizing two parameters. However, daily changes in confirmed cases do not follow a perfect Gaussian. Therefore, while fitted Gaussian fades away quickly, daily changes have oscillations on the right-hand side tail caused by the dynamic nature of the pandemic.

### E. Understanding the Effect of History By Analyzing the Short-Term Memory Weight Vector

It is important to understand the effect of the previous number of cases on current cases to assess the severity and the future of a pandemic. Analyzing the past helps to understand the net incubation period, the effect of regulations against the pandemic, whether the virus mutates or not, and the effect of a particular collective behavior in a country. Therefore, in this section, we analyze the memory property through optimized coefficients of previous time instances. As explained in Section II, at any time instant, we can express a function $g(x)$ as a weighted summation of its previous values and its derivative:

\begin{equation*} g\left ({x }\right)\cong \sum \limits _{k=1}^{l} \alpha _{k} g\left ({x-k }\right)+\sum \limits _{k=1}^{l} \beta _{k} g^{\prime }(x-k)\tag{47}\end{equation*}

Here, the second term in equation (47) can be expanded as:

\begin{align*} \sum \limits _{k=1}^{l} \beta _{k} g^{\prime }\left ({\text {x-k} }\right)= \sum \limits _{k=1}^{l} {\left [{ \beta _{k}g\left ({x+1-k }\right)-\beta _{k}g(x-k) }\right]} \\\tag{48}\end{align*}

To directly see the effect of any previous instant, we substitute (48) into (47). In this case, $\boldsymbol {g(x)}$ becomes:

\begin{equation*} g\left ({x }\right)=\sum \limits _{k=1}^{l} {A_{k}g(x-k)}\tag{49}\end{equation*}

where the weight coefficient of any previous instant $\boldsymbol {A}_{ \boldsymbol {k}}$ is calculated as follows:

\begin{equation*} A_{k}=\frac {1}{1-\beta _{1}}(\alpha _{k}-\beta _{k}+\beta _{k+1})\tag{50}\end{equation*}

In equation (49), $\boldsymbol {l}$ determines the number of previous steps considered in the modeling. The coefficients $\boldsymbol {\alpha }_{ \boldsymbol {k}}$ and $\boldsymbol {\beta }_{ \boldsymbol {k}}$ are found using the Taylor expansion, fractional derivative, and Least Square Method as explained in section II. Once these coefficients are found, the effect of any previous time instant can be understood by calculating $\boldsymbol {A}_{ \boldsymbol {k}}$ by equation (50). Together with $\boldsymbol {l}$ steps considered in the past, $\boldsymbol {A}_{ \boldsymbol {k}}$ coefficients form an $\boldsymbol {l}$-dimensional memory weight vector $\boldsymbol {H}$:

\begin{equation*} \boldsymbol {H}=[A_{1},A_{2},\ldots,A_{3}]\end{equation*}

Any element $\boldsymbol {A}_{ \boldsymbol {k}}$ of $\boldsymbol {H}$ represents how important the $\boldsymbol {k}^{ \boldsymbol {th}}$ previous step in regards to the current instance. To achieve better modeling and inference, experiments are carried out with $M=50$. In this section, normalized $\boldsymbol {H}$ vectors are analyzed using wavelet-based denoising and correlation coefficients.

**FIGURE 7.**
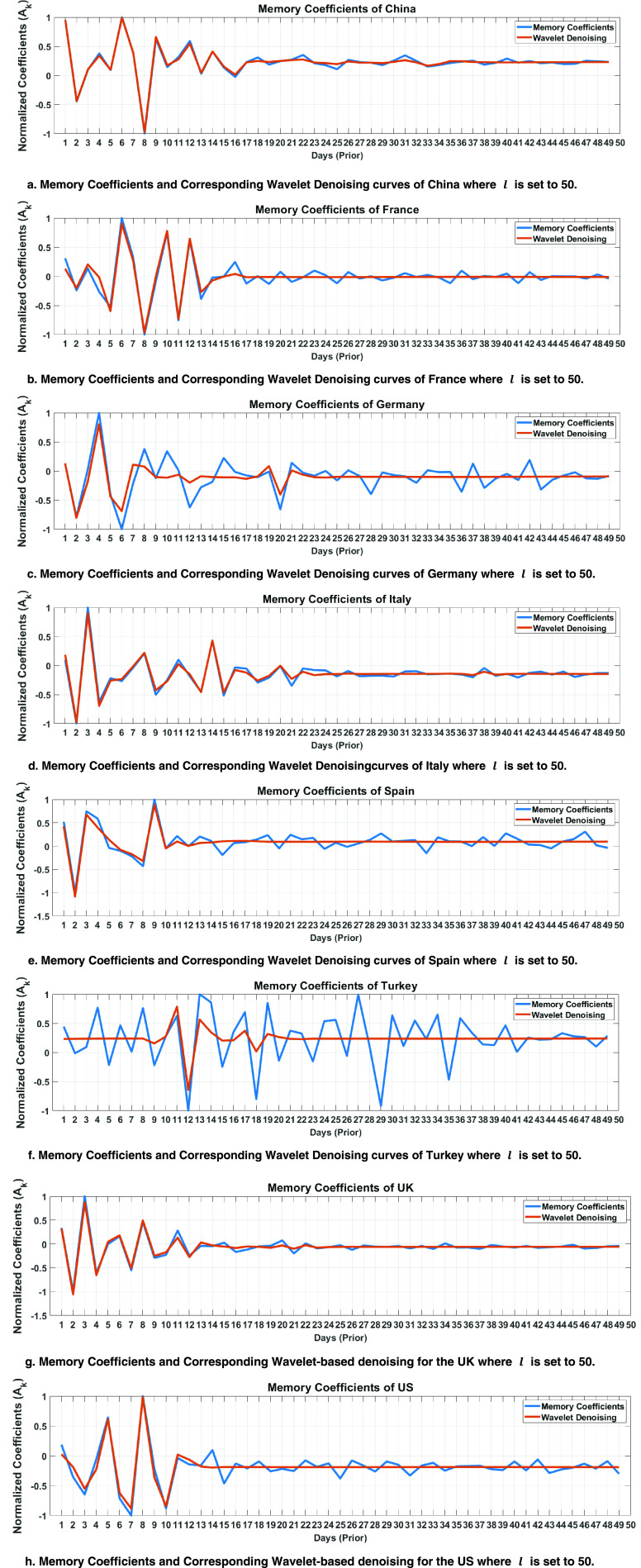
Normalized memory coefficients and corresponding wavelet-based denoised signals for China, France, Germany, Italy, Spain, Turkey, U.K., and the U.S.

Fig. 7 illustrates the normalized memory vectors for China, France, Germany, Italy, Spain, Turkey, the UK, and the US. To eliminate oscillations, wavelet-based denoising is applied to each memory vector, $\boldsymbol {H}$. For each figure, the blue curve denotes the original memory signal, while the red curve denotes the denoised signal. Figures indicate that, for all countries, except Germany and Turkey, coefficients after day 14–20 washes out and has oscillations. When wavelet-based denoising is applied, coefficients after 14–20 days are zeroed out. This means that any time instant can be mainly expressed in terms of the last two weeks. This is consistent with the incubation period of COVID-19, the number of confirmed cases today are mainly dependent on the number of cases in the last 14-days [1]. In countries like Germany, Italy, and the UK, the highest memory coefficients are observed within the first week. In the US and France, the highest peaks of coefficients span the second week. Based on this observation, Germany, Italy, and the UK have shorter average incubation periods when compared to the US and France.

Among all the countries, Turkey is the one with the largest non-zero memory period. In contrast to other countries, memory coefficients of Turkey include non-zero values up to the 40^th^ day. Considering COVID-19 has an incubation period up to 14 days, the reason behind this figure can be the noise on the existing data, the strategy against the pandemic, and the collective behavior of the country.

**FIGURE 8.**
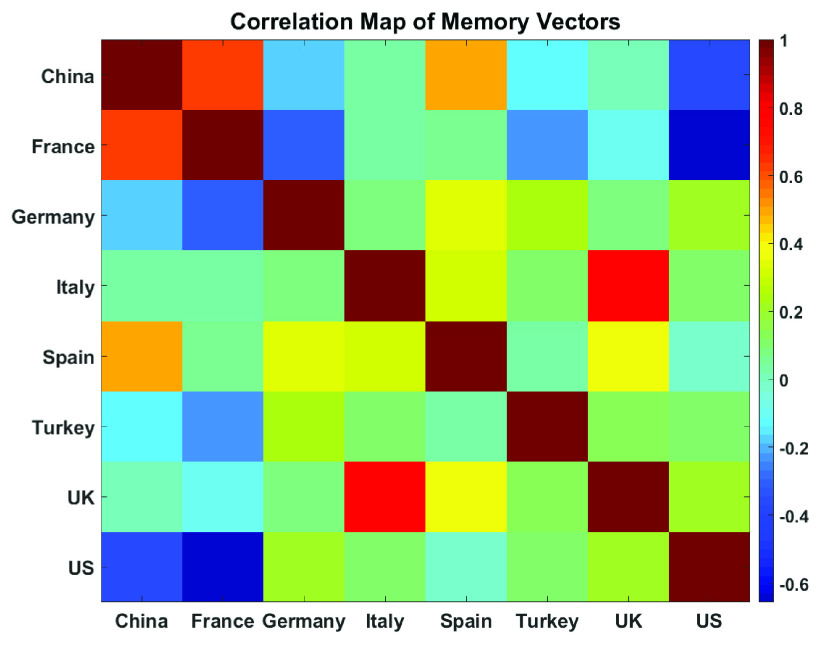
Correlation Coefficient, $R$, of Memory vectors between 8 countries: China, France, Germany, Italy, Spain, Turkey, U.K., and the U.S.more explicit integration into the modeling.

Fig. 8 shows the correlation coefficients (given in Table 6) between memory vectors by a color map. Correlation coefficient $\boldsymbol {R}$ takes values between [−1, 1] and the sign of $\boldsymbol {R}$ indicates the direction of a relationship. Positive $\boldsymbol {R}$ means that, if any value for a country increases, the other country also increases. Negative $\boldsymbol {R}$ means that if the value of one country increases, the value of the other country decreases. In this figure, the red color indicates a strong positive correlation while blue color indicates a strong negative correlation. The color bar is given on the right-hand side of the figure. One can see that the strongest correlation is observed in the UK– Italy pair, while the strongest negative correlation is observed for France and the US. France is negatively correlated with Germany and Turkey while China possesses highly positive correlations with France and Spain. Both China and France has a strong negative correlation with the US. On the other hand, the US and Germany do not have any positive correlation with another country, while Turkey does not maintain any relevant affair at all. Also, Spain has weak positive correlations with the UK, Germany, and Italy. Germany is negatively correlated with France. Lastly, two countries that have a strong positive correlation between them, the UK and Italy, have a similar pattern of correlation coefficient values. Both countries have a weak positive correlation with China, France, and Germany.

**TABLE 6:** Correlation Coefficients Between Country Pairs

Correlation Coefficient, R	China	France	Germany	Italy	Spain	Turkey	UK	USA
China	1	0.62640685	−0.1795882	0.02134012	0.50360116	−0.1232921	0.01180925	−0.3709889
France	0.62640685	1	−0.3059618	0.02498707	0.04795931	−0.2380957	−0.0916445	−0.6562742
Germany	−0.1795882	−0.3059618	1	0.08040289	0.34262272	0.24667393	0.07811075	0.20395324
Italy	0.02134012	0.02498707	0.08040289	1	0.30283785	0.11679214	0.79029593	0.0983885
Spain	0.50360116	0.04795931	0.34262272	0.30283785	1	0.03679665	0.35842576	−0.0286716
Turkey	−0.1232921	−0.2380957	0.24667393	0.11679214	0.03679665	1	0.12739537	0.10123804
UK	0.01180925	−0.0916445	0.07811075	0.79029593	0.35842576	0.12739537	1	0.21701357
US	−0.3709889	−0.6562742	0.20395324	0.0983885	−0.0286716	0.10123804	0.21701357	1

## IV. Limitations of the Study

In this study, the curve of daily changes is assumed to be a Gaussian function, however, in reality, it does not follow a perfect Gaussian for every country. Also, the model relies on the data without considering any prevention measures taken by governments and social distancing policies directly. Investigating the direct effect of preventions are left for future study. However, daily confirmed cases, active cases, and deaths show the strategies, politics, and preventions such as curfews, wearing masks, social distancing of each country indirectly. The change in the daily confirmed cases, active cases, and deaths overtime inherently keep the information about each country. Further, the time-dependent SIR model takes the dynamics of pandemic and thereby the effectiveness of government interventions over time into account, still, the impact e.g. of the reproduction factor (R-Value) needs a

## V. Conclusion

This document is aimed to provide practical suggestions on how to predict case numbers for a better strategy for planning health resources for patients under the current pandemic conditions. This modeling is not only for today’s COVID-19 reality, but it can also be used for future local or worldwide outbreaks. In this study, the number of confirmed cases, deaths, and recoveries of the COVID-19 outbreak is modeled and predicted for 8 countries including China, France, Germany, Italy, Spain, Turkey, the UK, and the USA.

First, we modeled the COVID-19 data, from the first confirmed case date to the 19^th^ of April by using our previously presented Deep Assessment Methodology which relies on Fractional Calculus. Then, a one-step prediction was made using the DAM and Long-Short Term Memory (LSTM) to assess the performance of DAM. The third part of the study focused on the short-term prediction of the pandemic where the following 30 days are predicted with the Time-Dependent SIR model and Gaussian model that relies on the derivative of the continuous number of confirmed cases obtained from DAM. The purpose of the Gaussian modeling was to predict the future of the pandemic through the daily changes of pandemic data and to estimate the peak number of cases. Employed Time-dependent SIR model effectively predicted the number of infected and recovered cases in the future up to the 19th of July. We showed that DAM successfully modeled the COVID19 dataset with 0.6671%, 0.6957%, and 0.5756% average MAPEs for confirmed cases, deaths, and recoveries, respectively. Also, results illustrated that DAM is superior to LSTM for a one-step prediction of the pandemic. Based on the analysis, it can be seen that fitting a Gaussian function on the dataset underestimates the future trend of the pandemic. The proposed Gaussian prediction method can be used in peak prediction, however, underestimates the future. Better prediction results can be achieved by taking the different distributions (generalized inverse Gaussian, Maxwell-Boltzmann, Nakagami, F and Fréchet distributions) into account that model the daily change of the number of patients for the future studies.

Lastly, an analysis of the past is made by applying wavelet-based denoising on memory coefficient vectors and calculating correlation coefficients between countries’ vectors obtained with the DAM. The experiments showed that, for all countries except Turkey, the current number of confirmed cases of the pandemic was mainly determined by the last 14 days which was consistent with the incubation period of COVID-19. Results point out that, countries like Germany, Italy, and the UK have a shorter average incubation period when compared to the US and France.

Evaluation of multivariable and multifunctional problems, analyzing time windows, randomness, noise, and error changes are also left to future work. Implementations of the DAM and Gaussian Prediction are publicly available at [57] and [58].

*Conflicts of Interest:* The authors declare no conflict of interest. The funders had no role in the design of the study; in the collection, analyses, or interpretation of data; in the writing of the manuscript, or in the decision to publish the results.
